# Diagnostic accuracy of ^13^N-ammonia PET, ^11^C-methionine PET and ^18^F-fluorodeoxyglucose PET: a comparative study in patients with suspected cerebral glioma

**DOI:** 10.1186/s12885-019-5560-1

**Published:** 2019-04-08

**Authors:** Qiao He, Linqi Zhang, Bing Zhang, Xinchong Shi, Chang Yi, Xiangsong Zhang

**Affiliations:** 1 Department of Nuclear Medicine, the First Affiliated Hospital of Sun Yat-sen University, 58# Zhongshan Er Road, Guangzhou, Guangdong Province 510080 People’s Republic of China; 2 Department of Nuclear Medicine, Affiliated Cancer Hospital&Institute of Guangzhou Medical University, Guangzhou, 510095 People’s Republic of China

**Keywords:** Glioma, ^13^N-NH_3_, ^18^F-FDG, ^11^C-MET, PET

## Abstract

**Background:**

The treatment of patients with glioma depended on the nature of the lesion and on histological grade of the tumor. Positron emission tomography (PET) using ^13^N-ammonia (NH_3_), ^11^C-methionine (MET) and ^18^F-fluorodeoxyglucose (FDG) have been used to assess brain tumors. Our aim was to compare their diagnostic accuracies in patients with suspected cerebral glioma.

**Methods:**

Ninety patients with suspicion of glioma based on previous CT/MRI, who underwent NH_3_ PET, MET PET and FDG PET, were prospectively enrolled in the study. The reference standard was established by histology or clinical and radiological follow-up. Images were interpreted by visual evaluation and semi-quantitative analysis using the lesion-to-normal white matter uptake ratio (L/WM ratio).

**Results:**

Finally, 30 high-grade gliomas (HGG), 27 low-grade gliomas (LGG), 10 non-glioma tumors and 23 non-neoplastic lesions (NNL) were diagnosed. On visual evaluation, sensitivity and specificity for differentiating tumors from NNL were 62.7% (42/67) and 95.7% (22/23) for NH_3_ PET, 94.0% (63/67) and 56.5% (13/23) for MET PET, and 35.8% (24/67) and 65.2% (15/23) for FDG PET. On semi-quantitative analysis, brain tumors showed significantly higher L/WM ratios than NNL both in NH_3_ and MET PET (both *P* < 0.001). The sensitivity, specificity and the area under the curve (AUC) by receiver operating characteristic (ROC) analysis, respectively, were 64.2, 100% and 0.819 for NH_3_; and 89.6, 69.6% and 0.840 for MET. Besides, the L/WM ratios of NH_3_, MET and FDG PET in HGG all significantly higher than that in LGG (all *P* < 0.001). The predicted (by ROC) accuracy of the tracers (AUC shown in parentheses) were 86.0% (0.896) for NH_3_, 87.7% (0.928) for MET and 93.0% (0.964) for FDG. While no significant differences in the AUC were seen between them.

**Conclusion:**

NH_3_ PET has remarkably high specificity for the differentiation of brain tumors from NNL, but low sensitivity for the detection of LGG. MET PET was found to be highly useful for detection of brain tumors. However, like FDG, high MET uptake is frequently observed in some NNL. NH_3_, MET and FDG PET all appears to be valuable for evaluating the histological grade of gliomas.

## Background

Primary malignant central nervous system (CNS) tumors represent approximately 2% of total cancers but account for high patient morbidity and mortality, especially in children and adolescents, which pose specific diagnostic and therapeutic challenges to neurologists [1]. Gliomas are the most common primary brain tumors, representing about 81% of all primary malignant CNS neoplasms [2]. The treatment of patients with brain glioma depended on the nature of the lesion and on the accurate pretherapy tumor grading. Brain lesions have overlapping imaging features on conventional computed tomography (CT) or magnetic resonance imaging (MRI) which are often difficult to make an accurate diagnosis. The interruption of blood brain barrier (BBB) is easily detected on contrast-enhanced MRI and CT which can provide useful information in the diagnosis of brain tumors, such as glioma, meningiomas and lymphoma. However, tumors with intact BBB like low-grade gliomas (LGG) may mimic non-neoplastic lesions (NNL) in morphological neuroimaging modalities. Besides, morphological evaluation is still difficult to assess accurately the tumor grade and the extent of the tumor invasion [3, 4]. Advanced MRI techniques can gain some valuable biochemical information by detecting the concentration of certain metabolites, but its clinical utility is still limited [5, 6]. Positron emission tomography (PET) is a molecular imaging technique allowing in vivo quantitative measurement of biological processes non-invasively, has become an integral part in the diagnostic workup of patients with brain lesions [7, 8].

^18^F-fluorodeoxyglucose (FDG) is an ^18^F-labeled glucose analog and has been used to measure glucose metabolism in vivo. Within this context, FDG PET has been commonly used in tumor detection, prediction of malignancy grade, evaluation of therapeutic response, and discrimination between radiation necrosis and tumor recurrence and between benign and malignant lesions [9–11]. However, FDG PET has limitations in the evaluation of brain tumors, due to the high-glucose metabolism in normal brain parenchyma and nonspecific uptake by NNL. ^11^C-methionine (MET), reflecting amino acid active transport and protein synthesis, is considerably more sensitive than FDG PET for the detection and delineation of brain tumors [12–14]. However, increased uptake of MET has also been reported in lots of NNL [15–17], prompting efforts to develop new oncological PET tracers. Since 2006, we have reported the clinical usefulness of ^13^N-ammonia (NH_3_) PET imaging for brain tumors, especially for gliomas, through a series of studies. The results showed that NH_3_ was a valuable PET tracer to differentiate gliomas from NNL, and to evaluate the histological grade of gliomas [18–22]. Since clinical application of NH_3_ PET have been restricted to just a few institutions, little information is available on the clinical utility of NH_3_ PET in the evaluation of brain tumors as compared with FDG PET and MET PET. The purpose of this study was to prospectively investigate the clinical potential of NH_3_ PET for the diagnosis of brain tumor and differentiating high-grade gliomas, in comparison with MET PET and FDG PET.

## Methods

### Patients

Ninety patients with suspicion of cerebral gliomas were prospectively enrolled in the study between September 2010 and December 2017. All patients who were referred for PET had an inconclusive diagnosis on previous conventional imaging (CT/MRI), and PET was performed to determine further diagnostic and therapeutic approach. NH_3_ PET, FDG PET and MET PET were performed within 1 week of each other. Patients remained untreated until the PET study completed. The reference standard was established by histology or clinical and radiological follow-up for at least one year. Histopathological diagnosis, based on the 2016 World Health Organization (WHO) classification, was obtained by operation or stereotactic brain biopsy. We grouped grade III and IV gliomas together as high-grade gliomas (HGG) and grade II and I together as LGG. The study was approved by the hospital ethics committee, and all patients gave their written informed consent before their participation in the study.

### Radiotracer synthesis

Tracers were produced at our center by commercially available system for the isotope generation (Ion Beam Applications; Cyclone-10, Belgium). NH_3_ was synthesized according to our published method [18]. MET was produced by the method of Chung et al. [14] and FDG was synthesized by the method of Toorongian et al. [23]. The radiochemical purity of NH_3_ and MET were > 99% and FDG was > 95%.

### PET imaging protocol

All patients were normoglycemic and had fasted for at least 6 h before FDG PET examination. No special dietary instructions were given to the patients before NH_3_ PET and MET PET examination. PET/CT imaging was acquired with a Gemini GXL 16 scanner (Philips, Netherlands) in 3-dimensional acquisition mode. A dose of 5.18 MBq (0.14 mCi)/kg FDG was injected intravenously and serial scanning was performed, approximately 30 min after the injection with the patient supine, resting, with their eyes closed. In the PET/CT system, non-contrast CT scan was acquired on the dual-slice spiral CT using a slice thickness of 3 mm, a pitch of 1 and a matrix of 512 × 512 pixels. After the CT scan, static brain PET acquisition was performed for 10 min per bed position for one bed with a matrix of 128 × 128 pixels and a slice thickness of 1.5 mm. For NH_3_ and MET PET, after intravenous injection of 7.4 MBq (0.20 mCi)/kg of NH_3_ or MET, patients rested in a quiet room and PET was performed 5 min later. The acquisition parameters were the same as that for FDG PET. Finally, PET images were reconstructed by LOR-RAMLA algorithm with low-dose CT images for attenuation correction.

### PET image analysis

PET images were interpreted by two experienced nuclear physicians independently, who were blinded to the clinical and anatomical imaging findings and reached a consensus. The tracer uptake of the lesion was evaluated by both visual evaluation and semi-quantitative analysis.

For visual analysis, the degree of tracer uptake by the lesion was visually classified into 3 grades as follows: reduced uptake (−); normal uptake (+−); increased uptake (+), as compared with tracer uptake by the contralateral or surrounding normal brain parenchyma.

For semi-quantitative analysis, a region of interest (ROI) was placed over the entire lesion on the transverse PET image, and the standardized uptake value (SUV) was calculated. For lesions with reduced or equal tracer uptake, the ROI was drawn based on the anatomical information on the brain lesions presented by MRI/CT. White-matter-ROI (10 mm in diameter) was drawn on the side contralateral to the tumor. L/WM ratio was determined by dividing the lesion (L) maximum SUV by the average SUV of the contralateral White-matter (WM).

### Statistical analysis

All the statistical analyses except the ROC analyses were performed using SPSS Statistics 17.0 (SPSS Inc. Chicago, IL, USA) software. Categorical data were expressed as numbers and frequency (%). The sensitivity, specificity, positive predictive value (PPV), negative predictive value (NPV), and accuracy of NH_3_, MET and FDG PET for the detection of brain tumor were calculated. Continuous variables are expressed as mean ± standard deviation (SD) and were compared using Student’s t-test. Receiver operating characteristic (ROC) curve was adopted to analyze the efficiency of L/WM ratios in the differential diagnosis among different groups. Further, the MedCalc statistics software was used to test for differences in ROC curve of the different. To determine whether tracer uptake of NH_3_, FDG and MET were related to each other, Spearman’s rank correlation coefficient was used. For all analysis, *P* value of less than 0.05 was considered statistically significant.

## Results

### Patients

Characteristics of all 90 patients are summarized in Table 1. Based on reference standard, 67 patients with brain tumor were all histopathologically confirmed by operation (*n* = 43) or stereotactic brain biopsy (*n* = 24). These 67 patients included 27 patients with LGG (4 WHO grade I and 23 WHO grade II), 30 patients with HGG (19 WHO grade III and 11 WHO grade IV), and 10 patients with non-glioma tumors. NNL was diagnosed in 23 patients. Further details of each individual patient with non-glioma tumors and NNL are given in Table 2. Among the 23 patients with NNL, 10 patients were histopathologically confirmed by operation (*n* = 3) or stereotactic brain biopsy (*n* = 7). The remaining 13 patients were diagnosed by clinical and radiological follow-up for at least one year (29 ± 5.4 months, range 15–47 months). Of them, 4 patients had demyelination or multiple sclerosis treated with corticosteroids, 6 patients had inflammation accepted anti-inflammatory therapies, 2 patients had infarction mainly accepted anticoagulant therapies, 1 patient had hemorrhage mainly treated with blood pressure management, all their neurological symptoms and MRI findings improved during follow-up.

**Table 1:** Characteristics of all patients

	All	LGG		HGG		Non-glioma tumors	NNL
N	90	27		30		10	23
Age range (mean ± SD, y)	3–78 (40.0 ± 14.0)	3–67 (34.0 ± 13.9)		14–61 (43.5 ± 12.6)		21–69 (42.0 ± 15.3)	22–78 (41.6 ± 14.0)
Sex (M: F)	3:2	19:8		16:14		3:7	16:7
Final diagnose		WHO	Grade I	WHO	Grade III		
		PA	4	AA	4		
		WHO	Grade II	AO	3		
		DA	1	AE	1		
		GA	1	Astrocytoma 11			
		OD	4	WHO	Grade IV		
		OA	1	GBM	11		
		Astrocytoma 16					

*LGG* low-grade glioma, *HGG* high-grade glioma, *NNL* non-neoplastic lesions, *PA* pilocytic astrocytoma, *DA* diffuse astrocytoma, *GA* gemistocytic astrocytoma, *OD* oligodendroglioma, *OA* oligoastrocytoma, *AA* anaplastic astrocytoma, *AO* anaplastic oligodendroglioma, *AE* anaplastic ependymoma, *GBM* glioblastoma

**Table 2:** Details of patient characteristics, results of each PET imaging, and reference criteria for the final diagnosis in patients with non-glioma tumors and non-neoplastic lesions

Pt. No.	Age range (y)	Location^a^	FDG		NH_3_		MET		Method of diagnosis	Final diagnosis
			Visual	L/WM^b^	Visual	L/WM	Visual	L/WM		
Non-glioma tumors										
1	50–55	Cerebellum	(+)	2.24	(+)	1.72	(+)	2.89	Operation	Metastatic adenocarcinoma
2	20–25	L. basal ganglia region	(−)	1.32	(+)	2.67	(+)	3.24	Biopsy	Germinoma
3	35–40	R. cavernous sinus area	(+)	2.38	(+)	2.30	(+)	3.35	Operation	Squamous cell carcinoma
4	40–45	Suprasellar	(−)	1.07	(+)	2.42	(+)	2.28	Operation	Craniopharyngioma
5	65–70	R. parietal	(−)	1.37	(+)	3.75	(+)	2.72	Operation	Meningioma
6	60–65	Cerebellum	(−)	1.28	(+)	3.66	(+)	2.78	Operation	Hemangioblastoma
7	25–30	Cerebellum	(+)	2.61	(+)	2.00	(+)	2.00	Operation	Medulloblastoma
8	25–30	Cerebellum	(+)	2.32	(+)	2.40	(+)	2.24	Operation	Medulloblastoma
9	45–50	L. frontal	(+)	4.02	(+)	3.09	(+)	3.00	Operation	Metastatic tumor
10	30–35	R. frontal	(−)	1.22	(−)	1.60	(+−)	1.51	Operation	DNT
Non-neoplastic lesions										
11	20–25	L. frontal	(+)	2.75	(+−)	1.68	(+−)	1.58	Follow-up	Inflammation
12	40–45	L. frontal-parietal	(−)	1.76	(−)	1.10	(−)	1.00	Biopsy	Demyelination
13	30–35	R. parietal	(+−)	2.10	(+−)	1.64	(+)	2.27	Follow-up	Demyelination
14	25–30	R. basal ganglia region	(+−)	2.12	(+−)	1.72	(+−)	1.50	Biopsy	Demyelination
15	70–75	Cervical spinal	(+)	2.88	(+−)	1.70	(+−)	1.78	Follow-up	Inflammation
16	20–25	R. cerebellopontine angle	(−)	1.32	(+−)	1.63	(+−)	1.55	Follow-up	Demyelination
17	25–30	R. thalamus	(−)	1.80	(+−)	1.75	(+−)	1.77	Follow-up	Inflammation
18	40–45	R. parietal	(+)	2.23	(+−)	1.72	(+)	2.49	Biopsy	Inflammation
19	35–40	R. cerebellum	(+)	2.80	(−)	1.45	(−)	1.38	Follow-up	Inflammation
20	40–45	Brain stem, R. temporal	(−)	1.58	(−)	1.55	(+−)	1.54	Follow-up	Inflammation
21	40–45	L. pons	(−)	1.56	(−)	1.29	(−)	1.31	Follow-up	Infarction
22	50–55	L. temporal	(+)	2.49	(−)	0.94	(+)	2.67	Operation	Brain abscess
23	45–50	L. parietal	(+)	3.28	(−)	1.63	(+)	2.88	Operation	Brain abscess
24	40–45	R. frontal	(+)	3.80	(−)	1.58	(+)	3.04	Operation	Brain abscess
25	20–25	L. parietal	(−)	1.10	(−)	1.18	(−)	1.32	Biopsy	Demyelination
26	30–35	L. basal ganglia region	(−)	1.44	(−)	1.45	(−)	1.40	Follow-up	Multiple sclerosis
27	40–45	R. cerebellum	(−)	1.98	(+−)	1.79	(+)	2.60	Follow-up	Infarction
28	50–55	L. frontal	(+−)	2.10	(+−)	1.75	(+)	1.95	Biopsy	Inflammation
29	78–80	R. parietal	(−)	1.06	(−)	1.14	(+)	1.86	Follow-up	Hemorrhage
30	40–45	L. frontal and parietal	(+)	2.70	(+)	1.87	(+)	3.15	Biopsy	Inflammation
31	40–45	Pons	(−)	1.68	(−)	1.54	(−)	1.38	Follow-up	Brain abscess
32	50–55	R. basal ganglia region	(−)	1.58	(−)	1.02	(+)	1.81	Follow-up	Infarction
33	25–30	L. thalamus	(−)	1.52	(−)	1.44	(−)	1.15	Biopsy	Demyelination

^a^ *R* right, *L* left, ^b^ *L/WM* Lesion-to-white matte ratio, *DNT* dysembryoplastic neuroepithelial tumor

### Visual analysis

**Fig. 1:**
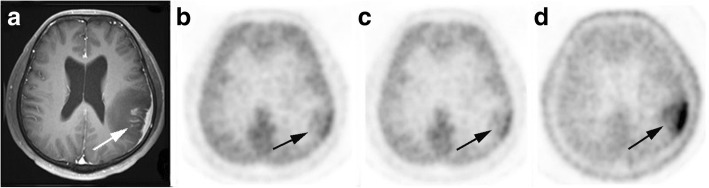
A patient aged 40–45 years with cerebral inflammatory lesion. Contrast enhanced MRI (**a**) shows the left frontal parietal lobe and adjacent meningeal lesion (arrow) was obvious heterogeneous enhanced. Significant higher uptake of ^11^C-MET (**d**) and mild increased uptake of ^18^F-FDG (**b**) and ^13^N-NH_3_ (**c**) were observed

Of the 67 patients with brain tumor, NH_3_ uptake was a visual grading of (+) in 42 patients (27 HGG, 6 LGG and 9 non-glioma tumor). Of the remaining 25 patients, 13 patients were (+−) and 12 patients were (−). It was interesting to note that, of the 23 patients with NNL, NH_3_ uptake was (+) only in one patient with inflammatory lesion, Fig. 1. Of the remaining 22 patients, 9 patients were (+−) and 13 patients were (−), Hence, NH_3_ was positive (+) in 43 patients (42 true positive and 1 false positive) and negative (+ − and -) in 47 patients (22 true negative and 25 false negative), detailed in Table 3. Sensitivity, specificity, PPV, NPV, and accuracy for the detection of brain tumor, using the visual grading criterion of (+), were 62.7, 95.7, 97.7, 46.8, and 71.1%, respectively.

**Table 3:** ^13^N-NH_3_, ^11^C-MET and ^18^F-FDG uptake positive in all lesion groups and mean L/WM ratios in neoplastic lesions, LGG, HGG and NNL

Group	No. of Patients	Positive, n			L/WM (mean ± SD)		
		NH_3_	MET	FDG	NH_3_	MET	FDG
Neoplastic lesions	67	42	63	24	2.25 ± 0.69	2.96 ± 0.95	2.00 ± 0.86
LGG	27	6	24	1	1.76 ± 0.51	2.34 ± 0.56	1.36 ± 0.35
HGG	30	27	30	18	2.59 ± 0.54	3.64 ± 0.90	2.59 ± 0.75
Non-glioma tumors	10	9	9	5			
NNL	23	1	10	8	1.50 ± 0.27	1.88 ± 0.63	2.07 ± 0.71

*LGG* low-grade glioma, *HGG* high-grade glioma, *NNL* non-neoplastic lesions, *L/WM* Lesion-to-white matter ratio

Of the 67 patients with brain tumor, MET uptake was a visual grading of (+) in 63 patients (all HGG, 24 LGG and 9 non-glioma tumor), and the remaining 4 patients were (+−). Of the 23 patients with NNL, MET uptake was (+) in 10 patients, and 6 patients were (+−) and 7 patients were (−). Hence, MET was positive in 73 patients (63 true positive and 10 false positive) and negative in 17 patients (13 true negative and 4 false negative). Sensitivity, specificity, PPV, NPV, and accuracy for the detection of brain tumor, using the visual grading criterion of (+), were 94.0, 56.5, 86.3, 76.5, and 84.4%, respectively.

**Fig. 2:**
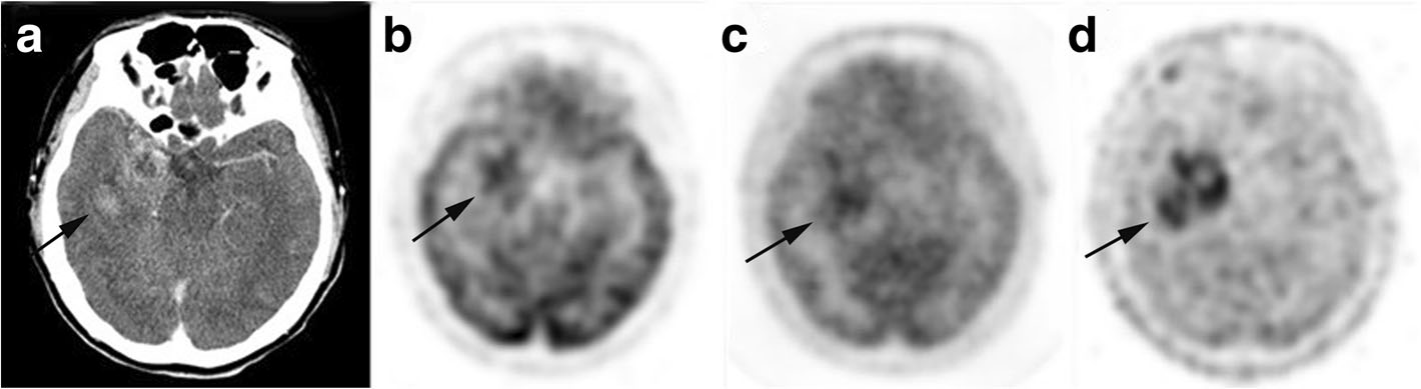
A patient aged 40–45 years with glioblastoma. Contrast enhanced CT (**a**) shows a moderately enhanced lesion in the right temporal lobe (arrow). ^18^F-FDG (**b**), ^13^N-NH_3_ (**c**) and ^11^C-MET (**d**) were all positive for the lesion, and the tracer accumulation of ^11^C-MET was higher than that of ^13^N-NH_3_ and ^18^F-FDG

**Fig. 3:**
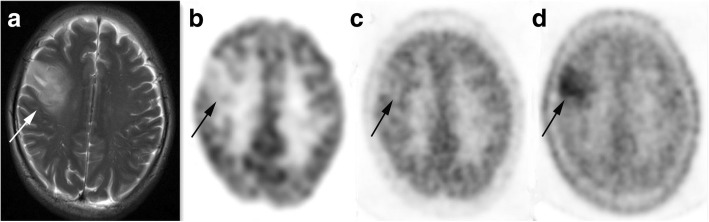
A patient aged 30–35 years with grade II glioma. T2-weighted MRI (**a**) shows a high signal lesion in the right frontal lobe (*arrow*). Higher uptake of ^11^C-MET (**d**), nearly equal uptake of ^13^N-NH_3_ (**c**) and apparently reduced uptake of ^18^F-FDG (**b**) were observed

**Fig. 4:**
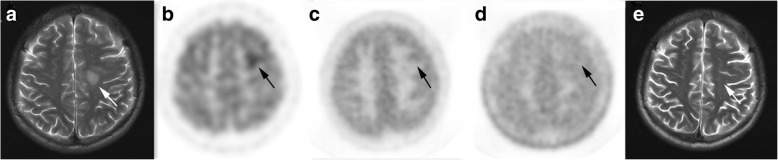
A patient aged 20–25 years with cerebral inflammatory lesion. T2-weighted MRI (**a**) shows a high signal lesion within the left frontal white matter (*arrow*). The lesion exhibits increased uptake of ^18^F-FDG (**b**) with equal uptake of ^13^N-NH_3_ (**c**) and ^11^C-MET (**d**) compared with contralateral normal brain parenchyma. Repeated MRI (**e**) shows that the lesion disappeared

**Fig. 5:**
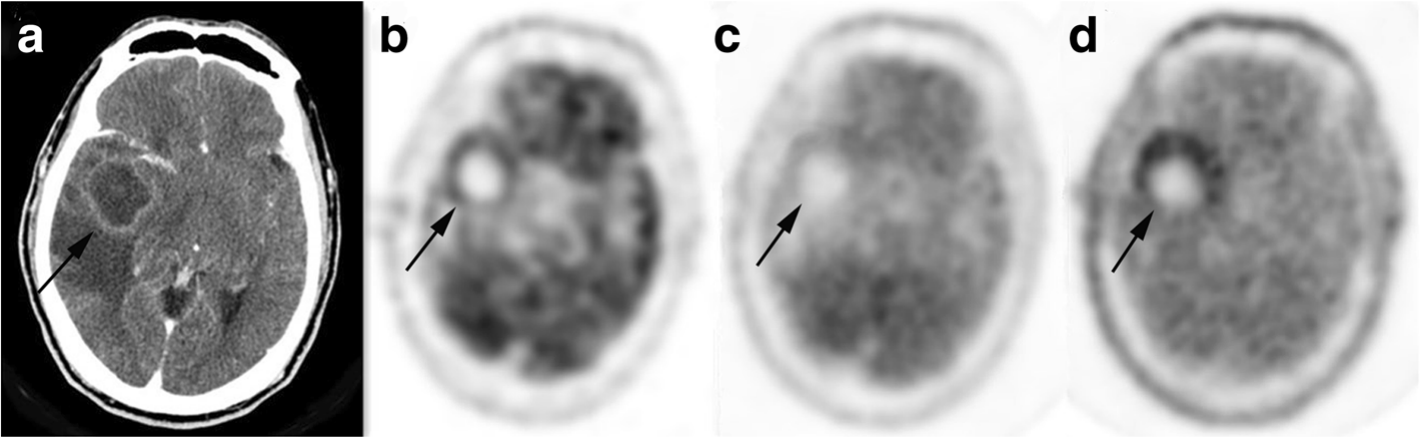
A patient aged 50–55 years with brain abscess. The lesion displays obviously increased uptake of ^11^C-MET (**d**) and mild increased uptake of ^18^F-FDG (**b**) with lower uptake of ^13^N-NH_3_ (**c**) compared with contralateral normal brain parenchyma. Contrast CT (**a**) shows a ring enhanced lesion in the right temporal lobe (arrow)

Of the 67 patients with brain tumor, FDG uptake was a visual grading of (+) in 24 patients (18 HGG, 1 LGG and 5 non-glioma tumor), and 5 patients were (+−) and 38 patients were (−). Among 23 patients with NNL, FDG uptake was (+) in 8 patients (all inflammatory or abscess lesion), and 3 patients were (+−) and 12 patients were (−). Hence, FDG was positive in 32 patients (24 true positive and 8 false positive) and negative in 58 patients (15 true negative and 43 false negative). Sensitivity, specificity, PPV, NPV, and accuracy for the detection of brain tumor, using the visual grading criterion of (+), were 35.8, 65.2, 75.0, 25.9, and 43.3%, respectively. Representative cases were shown in Figs. 1, 2, 3, 4, 5.

### Semi-quantitative analysis

Of the 90 patients (67 had brain tumors and 23 had NNL), the mean L/WM ratios of NH_3_ and MET PET in brain tumors were both significantly higher than that in NNL (2.25 ± 0.69 vs 1.50 ± 0.27, *P* < 0.001; 2.96 ± 0.95 vs 1.88 ± 0.63, *P* < 0.001), but not for FDG (2.00 ± 0.86 vs. 2.07 ± 0.71, respectively; *P* = 0.733) shown in (Table 3). ROC analysis for differentiation between brain tumors and NNL yielded an optimal L/WM ratio of 1.92 for NH_3_ (sensitivity, 64.2%; specificity, 100%; PPV, 100.0%; NPV, 48.9%; accuracy, 73.3%; AUC, 0.819; CI [0.734–0.904]), 1.97 for MET (sensitivity, 89.6%; specificity, 69.6%; PPV, 89.6%; NPV, 69.6%; accuracy, 84.4%; AUC, 0.840, CI [0.743–0.937]).

**Fig. 6:**
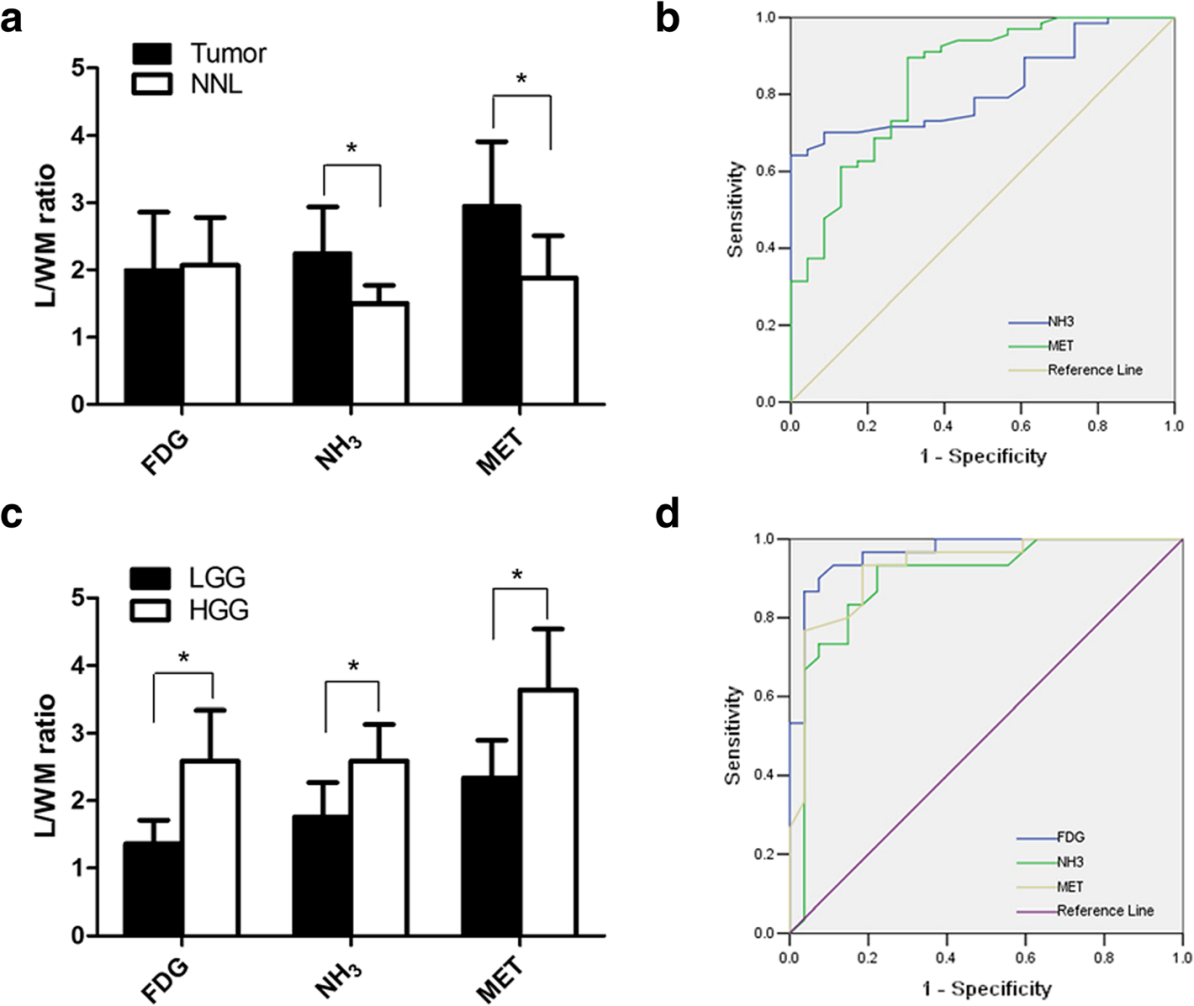
Graph (**a**) showing tracer uptake ratios in tumors and NNL. *P < 0.001. **b** ROC curve analysis of L/WM ratio to differentiate between tumors and NNL (NH_3_: AUC, 0.819; optimal cutoff, 1.92; MET: AUC, 0.840; optimal cutoff, 1.97). Graph (**c**) showing the correlation between tracer uptake and glioma grade. *P < 0.001. **d** ROC curve analysis of L/WM ratio to differentiate between HGG and LGG. Area under curve (AUC) was 0.964 for FDG (optimal cutoff, 1.65), 0.896 for NH_3_ (optimal cutoff, 2.01) and 0.928 for MET (optimal cutoff, 2.74)

Of the 57 patients with gliomas (27 had LGG and 30 had HGG), the L/WM ratios of NH_3_, MET and FDG PET in HGG were all significantly higher than that in LGG (2.59 ± 0.54 vs 1.76 ± 0.51, respectively, *P* < 0.001; 3.64 ± 0.90 vs 2.34 ± 0.56, respectively, *P* < 0.001; 2.59 ± 0.75 vs 1.36 ± 0.35, respectively, *P* < 0.001). (Table 3, Fig. 6a). ROC analysis for differentiation between HGG and LGG yielded an optimal L/WM ratio of 2.01 for NH_3_ (sensitivity, 93.3%; specificity, 77.8%; PPV, 82.4%; NPV, 91.3%; accuracy, 86.0%; AUC, 0.896; CI [0.807–0.986]), 2.74 for MET (sensitivity, 93.3%; specificity, 81.5%; PPV, 84.8%; NPV, 91.7%; accuracy, 87.7%; AUC, 0.928; CI [0.861–0.996]), and 1.65 for FDG (sensitivity, 93.3%; specificity, 92.6%; PPV, 93.3%; NPV, 92.6%; accuracy, 93.0%; AUC, 0.964; CI [0.920–1.000]), respectively. The greatest AUC was obtained for FDG uptake of the parameter L/WM ratio, but no significant differences were found. ROC curves of the three tracers were displayed in Fig. 6b.

### Correlation among three tracer accumulations

Significant correlation was observed among NH_3,_ MET and FDG uptake in gliomas (FDG/NH_3_: *r* = 0.726, FDG/MET: *r* = 0.762, NH_3_/MET: *r* = 0.776, all *P* < 0.001).

## Discussion

The major finding of our study is that NH_3_ PET could differentiate brain tumors from NNL with high specificity, compared with MET and FDG PET. It is interesting to note that only one of the 23 patients with NNL showed slightly high NH_3_ uptake. This finding suggested that NH_3_ had the potential to enable differentiation between neoplastic lesions and NNL, which was in good agreement with those reported in our previous studies [20]. However, in present study, NH_3_ PET detected only 62.7% (42/67) patients with brain tumor, and 77.8% (21/27) of patients with LGG, and 95.7% (22/23) of patients with NNL showed negative results in NH_3_ PET. This indicates that it is difficult to distinguish LGG from NNL using NH_3_ PET.

In the detection and delineation of glioma, MET has advantages over FDG, mainly due to its lower uptake in the normal brain tissue. In present study, for the detection of brain tumor, the overall ability of MET is better than NH_3_ and FDG, with a detection sensitivity of 94.0% versus 62.7% using NH_3_, and 35.8% using FDG. Given these results, MET PET appears to be superior to NH_3_ PET and FDG PET for detection of tumor lesions. However, increased uptake of these amino acid tracers is not specific as it has been observed in many NNL, including intracranial hemorrhages, cerebral infarctions, multiple sclerosis and brain abscess [16, 24–27]. In this study, MET PET revealed low specificity for brain tumors, as 10 of 23 NNL (6 patients with inflammatory and abscess lesions, 1 demyelination, 2 infarctions and 1 hemorrhage) showed MET uptake positive. Our results differ from Chung et al. report in benign brain lesions, probably because most of the benign lesions in our study were inflammatory lesions, but none or a small part in theirs study [14]. Mechanism of MET uptake in inflammatory lesions is not clear. One study showed that disruption of the BBB and increased density of inflammatory cells could account for this phenomenon [16].

According to the histological grade, in this present study when grade III and IV gliomas were grouped together as HGG and grade II and I together as LGG, NH_3_, MET and FDG uptake were all significantly higher in HGG (*n* = 30) than that in LGG (*n* = 27). These facts suggest that NH_3_, FDG and MET all have the potential to reflect degree of glioma differentiation. Some previous studies have also shown that the glucose utilization rate have a positive correlation with the malignancy grade of glioma [9, 10]. However, high FDG uptake has been also observed in NNL. These findings indicate that FDG PET may be not considered useful for differentiation between HGG and NNL. Although NH_3_ and MET PET seems to be valuable for evaluating the histological grade, the relationship between the tumor uptake of amino acid and histological grade remains controversial. Our findings are different from previous several studies [28–31]. In these studies, MET uptake seems to have no or less value for evaluating glioma malignancy grades. Ogawa et al. reported that there was a wide overlap of MET uptake between LGG and HGG despite statistically significant difference between them [32], thus they indicated that MET PET has greater value in assessing the extent rather than the histological grade of glioma. However, our results are consistent with a study by Singhal et al. who reported there was a significant higher uptake of MET in HGG than in LGG [33]. One of the reasons for the difference of these three radiopharmaceuticals uptake in gliomas may be the different mechanisms of accumulation in the tumor cells. The other interesting observation in our study is that the mean L/WM ratios for NH_3_, MET, and FDG PET have a statistically significant relationship to each other in cerebral gliomas. To our knowledge, this study is the largest series of head to head comparison among NH_3_, FDG and MET PET in patient with brain lesions.

## Conclusion

NH_3_ PET has remarkably high specificity for the differentiation of brain tumors from NNL, but low sensitivity for the detection of LGG. MET was found to be highly useful for detection of brain tumors. However, we should keep in mind that like FDG, high MET uptake is frequently observed in some NNL. NH_3_, MET and FDG PET all appears to be valuable for evaluating the histological grade of gliomas.

## Abbreviations

AUC: Area under the ROC curve
BBB: Blood brain barrier
CI: 95% Confidence interval
CT: Computed tomography
FDG: ^18^F-fluorodeoxyglucose
HGG: High-grade gliomas
LGG: Low-grade gliomas
MET: ^11^C-methionine
MRI: Magnetic resonance imaging
NH_3_: ^13^N-ammonia
NNL: Non-neoplastic lesions
NPV: Negative predictive value
PET: Positron emission tomography
PPV: Positive predictive value
ROC: Receiver operating characteristic
ROI: Region of interest
SUV: Standardized uptake value
